# Daily Cognitive Performance Under Stress: Contextual and Gender Differences

**DOI:** 10.1093/geroni/igaf122.184

**Published:** 2025-12-31

**Authors:** Nicole Richards, Elizabeth Munoz, Anqing Zheng, Sally Wadsworth, Martin Sliwinski, Chandra Reynolds

**Affiliations:** The University of Texas at Austin, Austin, Texas, United States; The University of Texas at Austin, Austin, Texas, United States; University of Colorado Boulder, Boulder, Colorado, United States; University of Colorado Boulder, Boulder, Colorado, United States; The Pennsylvania State University, University Park, Pennsylvania, United States; University of Colorado Boulder, Boulder, Colorado, United States

## Abstract

While exposure to stressors has been widely examined in the literature in relation to cognitive function, stress severity– the extent to which stressors are perceived as threatening or overwhelming– has emerged as a critical determinant of cognitive function and change. However, the momentary effects of stress severity on everyday cognitive performance remain unclear. This study examined the momentary associations between stress severity and cognitive performance using ecological momentary assessment data from the Colorado Adoption/Twin Study of Lifespan on behavioral development and cognitive aging. Participants (N = 440, Mage = 35.81, 57.7% women) completed momentary surveys and working memory tasks via smartphones up to three times daily for 14 days. Multilevel models, accounting for momentary and between-person confounders, revealed that people who tended to report greater stress severity had poorer working memory performance indexed by higher error scores (b = .009, CI[.004, .013], p < .001). At the momentary level, activities and gender moderated the momentary effect of stress severity on performance. Specifically, participants performed worse when they reported greater stress severity while “doing nothing” (b = .007, CI[.002, .012], p < .01), with this effect being more pronounced among women (b = .013, CI[.003, .022], p < .01). Conversely, for women, socializing buffered the negative impact of stress on cognitive performance (b = -.003, CI[-0.006, -0.001], p < .05). These findings highlight the importance of momentary context in understanding stress-related cognitive vulnerability and suggest that meaningful activities and social interactions may serve as protective factors, particularly for women.

